# Factors Influencing the Prognosis of Patients with Myalgic Encephalomyelitis/Chronic Fatigue Syndrome

**DOI:** 10.3390/diagnostics12102540

**Published:** 2022-10-19

**Authors:** Alaa Ghali, Carole Lacout, Jacques-Olivier Fortrat, Karine Depres, Maria Ghali, Christian Lavigne

**Affiliations:** 1 Department of Internal Medicine and Clinical Immunology, Angers University Hospital, F-49000 Angers, France; 2 Department of Vascular Medicine, Angers University Hospital, F-49000 Angers, France; 3 Department of General Medicine, Faculty of Medicine of Angers, University of Angers, F-49000 Angers, France

**Keywords:** myalgic encephalomyelitis/chronic fatigue syndrome, prognosis, diagnostic delay, age at disease onset

## Abstract

Myalgic encephalomyelitis/chronic fatigue syndrome (ME/CFS) is a long-term debilitating multisystem condition with poor prognosis. Studies that examined predictors of ME/CFS outcomes yielded contradictory results. We aimed to explore epidemiological and clinical prognostic factors of ME/CFS using operationalized criteria for recovery/improvement. Adult ME/CFS patients who attended the Internal Medicine Department of Angers University Hospital, Angers, France between October 2011 and December 2019, and were followed up until December 2020, were included retrospectively. Their medical records were reviewed for data collection. Patients were classified into two groups according to the presence or absence of recovery/improvement (R/I) and compared for epidemiological characteristics, fatigue features, post-exertional malaise severity, clinical manifestations, and comorbidities. The subgroups of recovered and significantly improved patients were then compared. 168 patients were included. Recovery and improvement rates were 8.3% and 4.8%, respectively. Older age at disease onset was associated with R/I (OR 1.06 [95% CI 1.007–1.110] (*p* = 0.028)), while diagnostic delay was inversely associated with R/I (OR 0.98 [95% CI 0.964–0.996] (*p* = 0.036)). The study findings confirmed the poor prognosis of ME/CFS and the deleterious effect of diagnostic delay on disease progression. Interestingly, being older at disease onset was associated with better outcomes, which offers hope to patients for recovery/improvement even at an advanced age.

## 1. Introduction

Myalgic encephalomyelitis (ME-ICC) [1], myalgic encephalomyelitis/chronic fatigue syndrome (ME/CFS) [2], and chronic fatigue syndrome (CFS) [3] are multisystem and long-term conditions of unknown etiology, and their pathophysiological mechanisms remain unknown [4]. These conditions share similar symptoms, including persistent or relapsing unexplained fatigue, post-exertional malaise (PEM), unrefreshing sleep, cognitive impairment, and musculoskeletal pain. Therefore, they severely impact quality of life and result in loss of productivity [5]. PEM is defined as the worsening of some or all of a patient’s baseline symptoms after exposure to a variety of stressors, including physical, mental, and emotional stressors that were normally tolerated before disease onset [1,2,4]. While PEM occurrence is not obligatory for the diagnosis of chronic fatigue syndrome [3], recent criteria consider PEM as a cardinal feature of the illness and require its presence [1,2,4]. The debate about whether ME, and CFS are distinct entities is still ongoing [4,6,7,8]. In this article, we will only use the term ME/CFS.

Studies that examined predictors of ME/CFS outcomes yielded contradictory results. This could be explained by the absence of operationalized criteria for recovery and improvement [9], the use of different diagnostic criteria, the duration of the illness, and the length of follow-up.

Although recovery and improvement rates varied widely across different studies, almost all studies agree that ME/CFS prognosis is rather unfavorable. The reported recovery rates ranged from 0% to 8% [5,10,11,12,13,14,15]. Likewise, a broad range of improvement rates from 17% to 64% was reported according to studies [5,10,13,15].

Another controversial issue is the age of disease onset and ME/CFS outcomes. While most research agrees that young people with ME/CFS have generally favorable prognosis [16,17], much uncertainty still exists about the relation between advanced age and poorer outcomes. Some studies [12,18,19] suggested that old age predicted persistent illness and poor prognosis, but others [11,20,21] did not find any link between age and poor outcomes. An improvement in the physical function of CFS patients over time, despite their age, has even been reported [22].

Similarly, a shorter duration of illness was reported to be a significant predictor of improvement in CFS patients [13,14,23,24,25], while other works failed to show this finding [11,21,26].

Moreover, fatigue severity at disease onset was suggested to be linked with poor outcomes [18,21,27,28]; however, this association was not consistent across all studies [15,16]. In parallel, PEM was found to be significantly associated with disability [25], perpetuated fatigue and predicted a poorer outcome for patients [29].

A worse prognosis was reported in ME/CFS patients with comorbid fibromyalgia [30], and some studies [16,25], but not others [13,19], showed that psychiatric comorbidity is predictive of poor CFS outcome.

Other factors, such as life stressors [31,32], female gender [32,33], and lower socioeconomic status [34] were also reported as being predictors of outcomes.

Death in patients with ME/CFS is usually caused by another co-existing pathology, such as cancer and cardiovascular diseases [35].

The purpose of this study was to explore epidemiological and clinical factors that could influence the prognosis of ME/CFS subjects.

## 2. Patients and Methods

### 2.1. Ethics

The study was approved by the ethics committee of Angers University Hospital (2020/147) and was conducted in compliance with the Helsinki Agreement. Data collection was approved by the French Data Protection Authority (CNIL).

### 2.2. Study Population

Adult ME/CFS patients aged 18 years and over diagnosed on the basis of the international consensus criteria (ICC 2011) [1], who attended the outpatient clinic of the Internal Medicine Department of Angers University Hospital, France between October 2011 and December 2019, and followed up until December 2020, were included retrospectively. Their medical records were reviewed for data collection.

### 2.3. Exclusion Criteria

Patients having medical conditions that may explain the chronic fatigue were excluded. In addition, patients with primary psychiatric disorders and those with substance dependency were also excluded. Fibromyalgia, irritable bowel syndrome (IBS), Hashimoto’s thyroiditis, and reactive depression were not considered exclusionary conditions as per ME-ICC 2011. Patients who had medical records with missing data and those who were lost to follow-up were not included in the study.

### 2.4. Study Design

Interviewing, history taking, physical examination, and follow-up of all included patients were carried out by the same physician.

#### Review of Patients’ Medical Records

The medical records of ME/CFS patients were carefully examined for the results of their initial assessment and the systematic follow-up visits. Data about epidemiological characteristics and comorbidities were gathered.

##### Initial Assessment

All included patients underwent a systematic 3-day hospitalization for an etiological workup and an overall assessment of fatigue levels and its impact on patient activities, as well as the frequency and severity of PEM and baseline manifestations.

##### Follow-Up Visits

The systematic bi-annual outpatient follow-up visits, which were held at the Department of Internal Medicine of Angers University Hospital, aimed at assessing self-reported changes, physical and cognitive fatigue levels, PEM severity, frequency and severity of symptoms, and the impact of the fatigue on patient activities, including occupational status. Recovered/improved patients continued to be followed up at the same frequency for self-reported changes, possible relapse of PEM or other symptoms, and occupational status.

### 2.5. Measures

The following dimensions were measured.

#### 2.5.1. Self-Reported Changes

At follow-up visits, the subjective experience of the personal situation was evaluated and patients were asked to describe changes in their current health state since the last assessment: recovered, improved, stationary, or worse.

#### 2.5.2. Fatigue Scales

The assessment of physical and cognitive fatigue and its severity was realized in all patients by means of validated self-reported questionnaires: the fatigue scale (FS) [36], and the fatigue severity scale (FSS) [37].

#### 2.5.3. Functional Impairment

The three subscales of the modified fatigue impact scale (MFIS) were used to assess physical, cognitive, and psychosocial functioning [38].

#### 2.5.4. PEM and Baseline Symptoms

The presence, frequency, and intensity of PEM and other illness-related symptoms over the past month were assessed by means of the standardized self-reported questionnaire of the Center for Disease Control and Prevention Symptom Inventory (CDC SI) [39].

#### 2.5.5. Occupational Status

At follow-up visits, patients were asked about their employment activity: return to work or not, number of hours spent working, including working from home and the travel time to and from the work site, early retirement due to illness, and receipt of disability pension.

### 2.6. Defining Recovery and Significant Improvement

The definition of both recovery and significant improvement made reference to the suggestion of Devendorf et al. [40], while also considering the absence of PEM as an obligatory criterion. In this way, patients were considered recovered if they were no longer experiencing PEM for at least 6 months, reported complete remission of their baseline symptoms, and were able to perform their premorbid levels of physical, cognitive, social, and occupational functioning without pacing strategies or taking medications. Patients were considered significantly improved if they were no longer experiencing PEM for at least 6 months, reported a substantial reduction in the number, frequency, or severity of their baseline symptoms, and were restoring certain levels of functioning in everyday activities, with a possible adherence to pacing strategies and/or medications.

### 2.7. Patients’ Grouping

We compared the proportion of recovered and significantly improved (R/I) patients with the rest of the study population who did not meet the criteria of recovery or significant improvement. We then analyzed separately and compared the subgroups of recovered (subgroup 1) and significantly improved patients (subgroup 2).

### 2.8. Statistical Analysis

Qualitative data were expressed as absolute number and percentage. Quantitative data were expressed as median and quartiles. Data were compared using the Chi-square test or Fisher’s test for qualitative data and the Mann–Whitney test or the Wilcoxon signed-rank test for quantitative variables. Multivariate analysis was performed with binary regression. The variables included in the model were those showing significant statistical difference in univariate analysis. A *p*-value of <0.05 was considered significant. The odds ratio (OR) was presented with its 95% confidence interval (CI). The analyses were performed using RStudio software v21.4.1106 (RStudio, Inc., Boston, MA, USA).

## 3. Results

**Figure 1:**
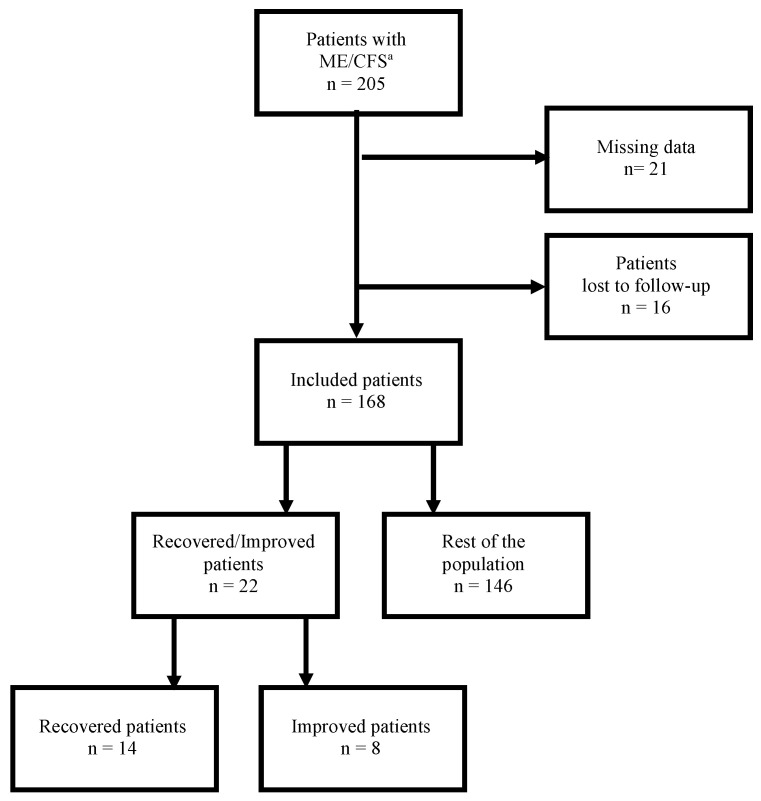
Flow chart of patients’ inclusion. ^a^ Myalgic encephalomyelitis/chronic fatigue syndrome.

Among the 205 patients who fulfilled the inclusion criteria, 21 had medical records with missing data, and 16 were lost to follow-up, so a total of 38 patients were excluded. Out of the 168 patients who were included in the study, 22 (13.1%) experienced recovery or significant improvement and constituted the group of R/I patients (Figure 1).

As summarized in Table 1, the comparison between the proportion of R/I patients and the rest of the study population showed no significant differences between both groups, except for the age at disease onset (*p* = 0.002), the diagnostic delay (*p* = 0.0004), the time of follow-up (*p* = 0.013), comorbid thyroiditis (*p* = 0.02), and comorbid irritable bowel syndrome (IBS) (*p* = 0.04).

The multivariate binary regression analysis showed that older age at disease onset was associated with R/I (OR 1.06 [95% CI 1.007–1.110] (*p* = 0.028)), while the diagnostic delay was inversely associated with R/I (OR 0.98 [95% CI 0.964–0.996] (*p* = 0.036)). A tendency towards a negative association between comorbid IBS and R/I was also observed (OR 0.35 [95% CI 0.090–1.087] (*p* = 0.090)) (Table 2).

In the R/I group, 14 patients were meeting recovery criteria with a return to normal premorbid functioning (subgroup 1) and 8 were experiencing significant improvement with restoration of certain levels of functioning in everyday activities (subgroup 2). Therefore, the recovery and significant improvement rates were 8.3% and 4.8%, respectively.

When taken together, the median age of patients at R/I was 46.3 [36.1–53.5] years, the median illness duration was 50 (31–62) months, and the median time from illness diagnosis to R/I was 17 [6.8–30] months. The median time of follow-up post R/I was 10 [12–35.8] months.

The comparison between both subgroups showed that they were comparable for all variables, especially the age at disease onset, the diagnostic delay, and the median time of follow-up post R/I (Supplementary Table S1).

The analysis of work-related outcomes showed that 5 (35.7%) patients in subgroup 1 were recovered 6–24 months after receiving financial compensation and 1 patient (12.5%) in subgroup 2 experienced significant improvement 6 months after being recognized as a disabled worker and receiving a disability pension (Table 3).

**Figure 2:**
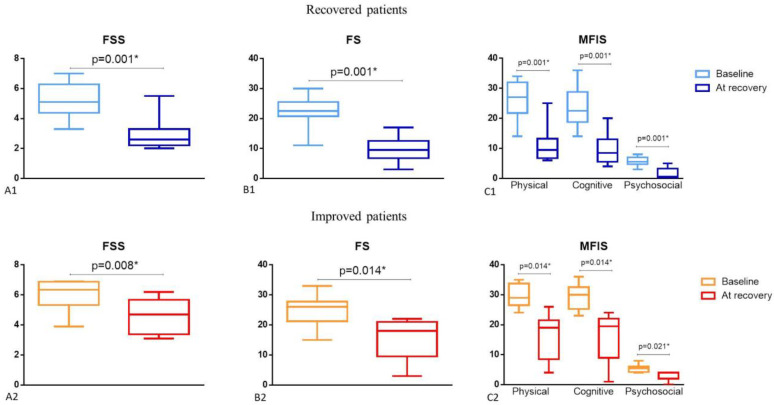
Fatigue evaluation of recovered (subgroup 1) and improved patients (subgroup 2) at baseline assessment and at improvement. FSS: Fatigue severity scale in recovered (A1) and improved patients (A2). FS: Fatigue scale in recovered (B1) and improved patients (B2). MFIS: Modified fatigue impact scale in recovered (C1) and improved patients (C2). * Statistically significant.

The evaluation of fatigue levels at recovery in subgroup 1 and at improvement in subgroup 2 found that FSS, FS, and MFIS median scores were significantly lower than those observed at initial assessment (Figure 2).

## 4. Discussion

Myalgic encephalomyelitis/chronic fatigue syndrome is a long-term and debilitating multisystem condition of unknown etiology, characterized by persistent or recurrent fatigue and the occurrence of PEM. ME/CFS leads to a significant reduction of premorbid levels of functioning of patients, resulting in deleterious effects on their employment, productivity, and overall quality of life. The prognosis of ME/CFS is globally poor as reported in the literature. The small number of studies on ME/CFS prognostic factors provided contradictory results. In this context, we conducted the present study to explore epidemiological and clinical factors that could influence the prognosis of ME/CFS subjects.

### 4.1. Recovery and Improvement Rates

In the current study, 100% of patients were followed up for 20 to 51 months (median 17 months) after the diagnosis was established. Out of the included 168 patients, 14 (8.3%) showed complete recovery, which is in line with previous reported recovery rates of 0–8%. [5,10,11,12,13,14,15]. However, the improvement rate observed among the study patients (4.8%) was much lower than that reported in the literature (17% to 64%) [5,10,13,15]. The large variability in recovery and improvement rates could be explained by the absence of operationalized criteria for recovery and improvement [40], the use of different diagnostic criteria, the duration of the illness, and the follow-up period.

### 4.2. Age at Disease Onset

In the current study, R/I patients had a significantly more advanced age at disease onset of 45 [32.5–48.3] years when compared to the rest of the population. The positive association between the age and a better outcome was confirmed by the multivariate binary regression analysis (OR 1.06 [95% CI 1.007–1.110] (*p* = 0.028)). This finding is in line with the study of Matthews and Komaroff [22] that observed an improvement in CFS patients overtime, despite the fact they were aging. On the other hand, our finding is not consistent with results from other studies that reported no association between age at disease onset and disease progression [11,20,21], and contradicts the findings of previous works suggesting that older age is a predictor of poor outcomes in adult ME/CFS patients [12,18,19].

This could be due to the fact that all study patients were asked to implement pacing strategies from the time of diagnosis and throughout follow-up in order to cope with their reduced energy levels [41]. These strategies consist of adapting and adjusting the patients’ various activities in terms of physical, cognitive, and emotional effort within the limits imposed by the illness. The physiological reduction in physical capacity with aging, in the absence of significant comorbidities, could make it easier for older people to apply pacing strategies and adjust activities according to their energy envelope. Consequently, a better prognosis was observed among this age range as compared to younger subjects.

Furthermore, from a professional point of view, it may be easier for older people to take early retirement that will allow them to adhere more closely to pacing strategies. For instance, in our study, a 54-year-old patient (*n* = 19) recovered 2 years after early retirement and was able to get another full-time job to increase his income (Table 3).

### 4.3. Diagnostic Delay

The diagnostic delay or illness duration corresponds to the time interval between the date of the onset of the symptoms and the date of the establishment of ME/CFS diagnosis. In the current study, R/I patients had a median diagnostic delay of 23 [16,17,18,19,20,21,22,23,24,25,26,27,28,29,30,31,32,33,34,35,36,37,38,39,40] months, which is significantly shorter compared to that of the rest of the study population, and in accordance with that reported in the study of van der Werf et al. [14], who found that all of the recovered patients had an illness duration of less than 1.5 years. Similarly, Reys et al. [23] concluded that the period of recovery was more likely in the early years of the illness. This difference persisted on multivariate binary regression analysis, which showed a negative association between diagnostic delay and R/I (OR 0.98 [95% CI 0.964–0.996] (*p* = 0.036)). This finding, suggesting that a shorter diagnostic delay predicts better outcomes in ME/CFS patients, is consistent with the results of prior studies, which observed that the improvement rate in patients with a long duration of complaints is small [13] and that longer illness duration is a significant predictor of poor prognosis [24,25,42]. Other works did not observe a relation between illness duration and prognosis [11,21,26].

The association of longer illness duration with a poor prognosis may be explained by the fact that longer-duration patients are less likely to experience spontaneous recovery [14] and more likely to develop cognitive decline [26], somatic complaints [42], allergies [26], and hypersensitivities [41]. Moreover, these patients are more susceptible to recurrent viral infections, which increase PEM severity and exacerbate baseline symptoms [43]. Comorbidities and reactive psychological distress that could occur over time may further worsen the prognosis.

### 4.4. Time of Follow-Up

Longer periods of follow-up were found to be associated with better outcomes [12,20]. In the current study, the median time of follow-up for R/I patients was significantly longer than that for the rest of the population but this difference did not persist on multivariate analysis, *p* = 0.12.

### 4.5. Baseline Fatigue Severity

Fatigue severity, which is generally associated with more severe illness, a greater number of physical symptoms, and an increase in functional limitations, was reported as a predictor of poor outcomes [18,21,27,28]. However, the associations of markers of a more severe illness with a poor outcome were not consistently found in the systematic review of studies on CFS prognosis [16]. Furthermore, although lower baseline fatigue scores were found to be linked to a better prognosis [13,14], this association was not consistent across all studies [15]. In the current study, all included patients suffered from high fatigue levels and fatigue-related impairments at baseline assessment. Nevertheless, the comparison between patients who experienced R/I and the rest of the study population found that the severity of fatigue and its impact were comparable between both groups. Likewise, PEM severity was not statistically different between both groups, in contrast with previous studies that found an association between PEM and poorer outcome [25,29].

### 4.6. Comorbidities

In the current study, a tendency towards poor outcomes was observed in ME/CFS patients with comorbid IBS (OR 0.35 [95% CI 0.090–1.087] (*p* = 0.090)) (Table 2). This finding is consistent with results from other studies that found higher levels of functional impairment in patients with both diseases compared to those who only have ME/CFS [44,45]. Furthermore, comorbid depression did not appear to be a predictor of a poor outcome, which is consistent with results of some studies [13,19] but not others [16,25]. In addition, contrary to the findings of Ciccone et al. [30], no association between comorbid fibromyalgia and poor outcome was observed in the current study.

### 4.7. Comparison between Recovered and Significantly Improved Patients

As expected, both recovered and improved patients experienced a significant reduction in their initial fatigue levels assessed by FSS and FS scales. In recovered patients, the FSS median score at initial assessment was 5.8 [5.1–6.8] vs. 3.3 [2.5–5.2] after recovery (*p* = 0.001), and the FS median score at initial assessment was of 25 [22.3–27] vs. 12 [8–16.3] after recovery (*p* = 0.001). Improved patients showed FSS median score at initial assessment of 5.2 [3.8–5.8] vs. 3.1 [2.1–3.1] after recovery (*p* = 0.008), and FS median score at initial assessment of 22 [18.8–25.3] vs. 9.5 [6.8–15] after recovery (*p* = 0.014). Initial fatigue-related impairment also improved significantly with a statistically significant reduction of physical, cognitive, and psychosocial MFIS scores (Figure 2).

These findings reflect the ability of R/I patients to perform all or a part of their premorbid levels of physical, cognitive, social, and occupational functioning, and confirm that these patients are fulfilling the criteria that we set up in the current study to define recovery or significant improvement.

The comparison between recovered and improved patients showed no statistically significant differences between both subgroups, especially the age at disease onset (36.5 [31.3–47.5] years for recovered patients vs. 43 [36.3–47.8] years for improved patients; *p* = 0.47), the diagnostic delay (16 [16.3–40] months for recovered patients vs. 26 [13–31.5] months for improved patients; *p* = 0.63), and the median time of follow-up post R/I (17.5 [15,16,17,18,19,20,21,22,23,24,25,26,27,28,29,30,31,32,33,34,35,36,37,38,39,40] months for recovered patients vs. 8.5 [11.3–25.5] months for improved patients; *p* = 0.18) (Supplementary Table S1). No relapses were observed during the follow-up period.

One question, however, remains unanswered: why some patients almost completely return to their premorbid levels of functioning while others restore only a part of their performances before illness. We could speculate that the heterogeneity of the illness may provide at least a partial answer. We know that many studies subtyped ME/CFS patients on the basis of various variables, especially biological ones. For instance, a subset of patients was found to display decreased metabolism due to mitochondrial dysfunction, and the extent of this dysfunction varied from one patient to another, and correlated significantly with ME/CFS severity [46,47]. Similarly, immune dysfunction with varying levels of pro-inflammatory cytokines was reported in a subset of patients suffering from various levels of fatigue and flu-like symptoms related to immune impairment [48]. Furthermore, mast cell activation syndrome [47] and/or postural orthostatic tachycardia syndrome [49], which are encountered in some ME/CFS patients, may be responsible for worsening of ME/CFS symptoms and/or the delay in recovery/improvement. Further studies are needed in order to elucidate this question.

### 4.8. Work-Related Outcomes

It is of interest to note that among the patients who recovered, 5 patients (35.7%) reported being cured 6–24 months after receiving financial compensation, including disability pension (3 patients), redundancy (1 patient), and early retirement pension (1 patient), and all of them were able to return to full-time work after recovering. In addition, 1 patient (12.5%) experienced significant improvement 6 months after being recognized as a disabled worker and receiving a disability pension, but he did not return to work (Table 3). In total, more than one fourth of R/I patients experienced better prognosis after receiving financial aid. This finding, which is in line with the results of a prior study [25], demonstrates that the receipt of long-term compensation providing a stable financial support could play a role in improving the prognosis of ME/CFS patients. On the contrary, financial difficulties linked to work inability seemed to be a risk factor for suicidal ideation in ME/CFS patients [50].

### 4.9. Strengths and Limitations

Although all study patients were asked to apply pacing strategies from the time of diagnosis and throughout follow-up, their adherence to such strategies was certainly not the same. This would therefore represent a bias in identifying prognostic factors.

Other limitations of this study include its retrospective character and the lack of information about fatigue scores in some patients. The MFIS scale that was not validated among ME/CFS patients was used concomitantly with two other scales, FS and FSS, as recommended by the CDC-NINDS project [51].

On the other hand, we would like to highlight the sizable number of the study population that included adult subjects who were examined and diagnosed by the same physician and underwent a same standardized procedure in terms of fatigue and PEM assessments. Diagnoses were established on the basis of the same criteria [1,4], and all patients were managed in the same way. The current study has the merit of defining recovery and significant improvement as well as differentiating between both concepts while taking into account the occupational dimension.

## 5. Conclusions

Studies that examined predictors of ME/CFS outcomes yielded inconsistent results, so we explored factors that could influence ME/CFS prognosis by using operationalized criteria for recovery and significant improvement. Our study revealed low recovery/improvement rates emphasizing the poor prognosis of ME/CFS patients. The analysis of the controversial role of age of onset as a prognostic factor found a statistically significant positive association between age at onset and better outcomes among ME/CFS patients. This finding could offer hope to older ME/CFS patients for the prospect of recovery or improvement with better quality of life, even at an advanced age. In addition, our analysis showed that diagnostic delay predicts poor prognosis for ME/CFS patients, which is consistent with the body of evidence in the literature. Unfortunately, almost all ME/CFS patients are un- or misdiagnosed for long periods, resulting in a long delay in time to diagnosis. This can be due to the heterogeneous non-specific ME/CFS symptoms and the lack of knowledge about ME/CFS among primary care physicians. Patients may consult many different specialists and undergo multiple explorations before ME/CFS diagnosis is made. In addition to the fact that waiting for diagnosis is always a worrying time for patients, diagnostic delay exacerbates, in most cases, the PEM as well as the baseline symptoms and negatively impacts the physical and mental state of patients. It therefore seems necessary to diagnose and manage people with ME/CFS as early as possible to prevent worsening of symptoms and improve prognosis. To achieve this, it is important to raise awareness among health professionals, especially primary care physicians about ME/CFS and PEM.

## Figures and Tables

**Table 1:** Comparison between myalgic encephalomyelitis/chronic fatigue syndrome patients with and without recovery/significant improvement.

	Patients with Recovery/Significant Improvement	The Rest of the Study Population	*p*
**Epidemiological characteristics**			
Patients, *n* (%)	22 (13.1)	146 (86.9)	
Female, *n* (%)	16 (72.7)	103 (70.5)	0.83
BMI, kg/m^2^	24.2 (21.4–26.7)	22.8 (20–26.2]	0.25
Age at data collection, years	49.1 (39.5–57.3)	43.8 (35.8–49.6)	0.07
Age at disease onset, years	45 (32.5–48.3)	32 (25–40)	0.002
Age at diagnosis, years	45.5 (34.3–51)	40 (32–46)	0.14
Diagnostic delay, months	23 (16–40)	55 (24–102)	0.0004
Time of follow-up, months	46 (34–56)	32.5 (19–49)	0.013
Family history of fatigue, *n* (%)	1 (4.5)	8 (5.5)	>0.99
Sudden onset of the illness, *n* (%)	14 (64.1)	74 (50.7)	0.26
Identified illness precipitants	18 (81.1)	107 (73.3)	0.39
Infectious precipitants	14 (64.1)	74 (50.7)	0.26
Non-infectious precipitants	4 (18.2)	33 (22.6)	0.79
**Baseline fatigue assessment**			
Fatigue severity scale	5.9 (5.2–6.4)	5.9 (5.2–6.5] (*n* = 130)	0.34
Fatigue scale	25 (20–27)	24 (21–28] (*n* = 119)	0.60
MFIS ^a^ physical	28 (26–32.8)	30 (26–33] (*n* = 119)	0.69
MFIS cognitive	25.5 (19.3–30.8)	29 (22–33] (*n* = 119)	0.12
MFIS psychosocial	6 (5.6)	6 (4–7] (*n* = 119)	0.52
**Baseline PEM ^b^ assessment**			
PEM frequency	3 (2.25–3)	3 (3–3)	0.90
PEM intensity	4 (4–4)	4 (2.5–4)	0.16
PEM severity	10 (8–12)	12 (7.5–12)	0.30
**Clinical manifestations, *n* (%)**			
Difficulty processing information	22 (100)	140 (95.9)	>0.99
Short-term memory loss	17 (77.3)	130 (89)	0.16
Headaches	16 (72.7)	106 (72.6)	0.99
Myalgia	20 (90.9)	127 (87)	>0.99
Arthralgia	13 (59.1)	96 (65.8)	0.54
Disturbed sleep patterns	19 (86.4)	123 (84.2)	>0.99
Unrefreshed sleep	20 (90.9)	140 (98.6)	0.28
Neurosensory and perceptual disturbances	18 (81.8)	132 (90.4)	0.26
Motor disturbances	21 (95.5)	136 (93.1)	>0.99
Flu-like symptoms	10 (45.5)	89 (61)	0.17
Recurrent infections	8 (36.4)	53 (36.3)	>0.99
Gastrointestinal impairments	20 (90.9)	118 (83.1)	>0.99
Urinary impairments	4 (18.2)	51 (36.1)	0.15
Orthostatic intolerance	6 (27.3)	62 (42.5)	0.18
Palpitation	14 (63.6)	94(64.4)	0.12
Vertigo	16 (72.2)	78 (53.4)	0.09
Respiratory involvement	10 (45.5)	82 (56.2)	0.35
Intolerance to extreme temperatures	13 (59.1)	103 (70.6)	0.28
**Comorbidities, *n* (%)**			
Reactional depression	8 (36.4)	52 (35.6)	0.95
Hashimoto’s thyroiditis	3 (13.6)	11 (7.5)	0.02
Fibromyalgia	1 (4.5)	31 (21.2)	0.08
Irritable bowel syndrome	5 (22.7)	59 (40.4)	0.04

Qualitative data were expressed as absolute number and percentage. Quantitative data were expressed as median and quartiles. ^a^ Modified fatigue impact scale. ^b^ Post-exertional malaise.

**Table 2:** Multivariate analysis of factors associated with recovery/significant improvement.

	Estimate	*p*-Value	OR (95% CI) ^a^
Intercept	−3.566	0.002	0.03 (0.002–0.252)
Age at onset	0.054	0.028	1.06 (1.007–1.110)
Diagnostic delay	−0.017	0.036	0.98 (0.964–0.996)
Time of follow-up	0.019	0.121	1.02 (0.995–1.044)
Thyroiditis	0.355	0.720	1.43 (0.160–8.895)
Irritable bowel syndrome	−1.052	0.090	0.35 (0.090–1.087)

Multivariate analysis was performed with binary regression. The variable to explain was recovery/significant improvement. The variables included were those showing significant statistical difference between patients with and without recovery/significant improvement (*p* < 0.05). ^a^ Odds ratio with 95% confidence interval.

**Table 3:** Work-related outcomes of recovered and improved patients.

N° ^a^	R/I ^b^	Age at R/I, Years	Work Status at Diagnosis	Work-Related Outcomes
1	I	61.6	Forklift operator on sick leave	Not able to return to working, but can do handiwork and gardening 4 h/day
2	R	46.6	Working as a cook. Part-time	Recovered 9 months after redundancy and returned to full-time work
3	R	30.5	Office work. Part-time	Returned to full-time work
4	R	30.5	Forklift operator. Part-time	Recovered after reducing hardship at work. Full-time work
5	I	50.0	Hairdresser. Part-time	Not able to return to working but can do sewing work 2 h/day
6	R	56.0	Nurse. Part-time	Returned to full-time work
7	R	34.0	Working in a fashion store. Part-time	Returned to full-time work
8	I	39.3	Working as a cleaner on sick leave	Professional retraining. Half-time work
9	R	34.5	Working in sales. Part-time	Returned to full-time work
10	R	42.3	Working in a bar on sick leave	Returned to full-time work
11	R	59.2	Plumber on sick leave	Recovered 9 months after being recognized as a disabled worker and returned to full-time work
12	R	46.0	Working in computer field. Part-time	Returned to full-time work
13	I	48.9	School teacher on sick leave	Part-time work
14	I	46.7	Working in computer field on sick leave	Improved 6 months after being recognized as a disabled worker (2nd degree invalidity). Not able to return to working
15	I	56.5	School teacher on sick leave	Not able to return to working
16	I	53.3	Assistant manager on sick leave	Not able to return to working
17	R	41.3	Working in a restaurant on sick leave	Returned to full-time work
18	R	60.3	Assistant nurse on sick leave	Recovered 10 months after being recognized as a disabled worker (2nd degree invalidity) and was able to get another full-time job
19	R	53.5	Manager in an educational service on sick leave	Recovered 2 years after early retirement and was able to get another full-time job
20	R	42.9	Project manager. Part-time	Returned to full-time work
21	I	32.3	Working in a museum on sick leave	Not able to return to working
22	R	35.1	Working in sales on sick leave	Recovered after being recognized as a disabled worker and was able to get another full-time job

^a^ Patient’s number; ^b^ recovery/significant improvement.

## Data Availability

The data presented in this study are available on request from the corresponding author. The data are not publicly available due to privacy restrictions.

## Supplementary Materials

The following supporting information can be downloaded at: www.mdpi.com/2075-4418/12/10/2540/s1, Table S1: Epidemiological characteristics, clinical manifestations, and comorbidities of recovered patients and significantly improved patients.
