# Supplementary material for: Factors Influencing the Prognosis of Patients with Myalgic Encephalomyelitis/Chronic Fatigue Syndrome
Source: Diagnostics (Basel). 2022 Oct 19;12(10):2540. doi: 10.3390/diagnostics12102540 (PMC9600584; doi:10.3390/diagnostics12102540)
Supplement: Supplementary file 1 [file diagnostics-12-02540-s001.zip › diagnostics-1952041-supplementary.pdf]

Table S1. Epidemiological characteristics, clinical manifestations, and comorbidities of recovered patients and significantly improved patients.

	Recovered patients *n* = 14	Significanlty improved patients *n* = 8	*p*
**Epidemiological characteristics**			
Female, *n* (%)	9 (64.3)	7 (87.5)	0.239
BMI, Kg/m^2	27 (22–27)	22.4 (20.1–26.2)	0.632
Age at data collection, years	42.3 (38.4–56)	48.3 (47.8–55.8)	0.339
Age at disease onset, years	36.5 (31.3–47.5)	43 (36.3–47.8)	0.47
Age at diagnosis, years	37.5 (33–49.5)	44.5 (41.8–52.3)	0.289
Diagnostic delay, months	16 (16.3–40)	26 (13–31.5)	0.632
Age at R/I[a], years	40.8 (34.7–51.8)	47.0 (44.8–54.1)	0.274
Time from onset to R/I, months	48 (28–63)	47.5 (35.5–58.5)	0.864
Time from diagnosis to R/I, months	32 (7.3–23)	21 (8.3–34.3)	0.516
Time of follow up, months	49.5 (40.5–55)	29.5 (32.3–56.3)	0.584
Time of follow up post R/I, months	17.5 (15–40)	8.5 (11.3–25.5)	0.180
Sudden onset of the illness, *n* (%)	7 (50)	7 (87.5)	0.167
Identified illness precipitants, *n* (%)	117 (78.6)	7 (87.5)	0.538
**Clinical manifestations, *n* (%)**			
Post exertional malaise severity	9.8 (8–12]	10 (8–13)	0.426
Difficulty processing information	14 (100)	8 (100)	NC
Short-term memory loss	14 (100)	7 (87.5)	0.363
Headaches	10 (71.4)	6 (75)	0.84
Myalgia	12 (54.5)	8 (100)	0.515
Arthralgia	8 (36.4)	5 ()	1.00
Disturbed sleep patterns	12 (85.7)	7 (87.5)	1.00
Unrefreshed sleep	14 (63.6)	6 (75)	0.121
Neurosensory and perceptual disturbances	11 (78.6)	7 (87.5)	1.00
Motor disturbances	13 (92.9)	8 (100)	1.00
Flu-like symptoms	6 (42.9)	4 (50)	0.759
Recurrent infections	4 (28.6)	4 (50)	0.418
Gastrointestinal impairments	13 (59.1)	7 (87.5)	
Urinary impairments	3 (21.4)	1 (12.5)	1.00
Orthostatic intolerance	3 (21.4)	3 (37.5)	0.624
Palpitation	10 (71.4)	4 (50)	0.386
Vertigo	10 (71.4)	6 (75)	0.840
Respiratory involvement	6 (42.9)	4 (50)	0.759
Intolerance to extreme temperatures	8 (57.1)	5 (62.5)	1.00
**Comorbidities, *n* (%)**			
Reactional depression	5 (35.7)	3 (37.5)	1.00
Thyroiditis	2 (14.3)	1 (12.5)	1.00
Fibromyalgia	1 (7.1)	0 (0.0)	1.00
Irritable bowel syndrome	4 (28.6)	1 (12.5)	0.60

Qualitative data were expressed as absolute number and percentage; Quantitative data were expressed as median and quartiles; [a]Recovery/significant improvement.
